# Exploring the Therapeutic Potential of High Dose Co-amoxiclav (1 gm) in Different Clinical Conditions: A Review

**DOI:** 10.7759/cureus.64717

**Published:** 2024-07-17

**Authors:** Debasis Jena, Tushar Kanti Ghosh, Amitrajit Pal, Dattatray Pawar, Akhilesh Sharma

**Affiliations:** 1 Otorhinolaryngology (ENT), Srirama Chandra Bhanja (S.C.B. Medical College &amp; Hospital, Cuttack, IND; 2 Ear, Nose, and Throat, Ghosh ENT Foundation, Kolkata, IND; 3 Medical Affairs, Alkem Laboratories Ltd., Mumbai, IND

**Keywords:** amoxicillin 875 mg, co-amoxiclav 1 gm, safety, clinical efficacy, infection

## Abstract

Amoxicillin/clavulanate (co-amoxiclav) is a widely used antibiotic in community healthcare settings, combining amoxicillin and clavulanate potassium to combat β-lactamase-producing bacteria. Despite its extensive use, limited pharmacokinetic/pharmacodynamic data support current dosing guidelines. This review explores the significance of high-dose co-amoxiclav (875 mg/125 mg) in treating various infections amidst rising antibiotic resistance. A comprehensive narrative literature review was conducted using MEDLINE, PubMed, and Google Scholar, focusing on co-amoxiclav 875 mg/125 mg from 1992 to 2024. Keywords included "Co-amoxiclav 875mg/125mg," "amoxicillin 875mg," "Co-amoxiclav dosing," "pharmacology," "PK," and "safety." Studies on non-safety aspects, those on cost-effectiveness, non-English articles, and those without full-text access were excluded. Clinical efficacy studies demonstrate the effectiveness of co-amoxiclav (875 mg/125 mg) in treating conditions such as cutaneous actinomycosis, actinomycetoma, lower respiratory tract infections, acute bacterial maxillary sinusitis, and community-acquired pneumonia. Comparative studies reveal similar or superior efficacy of co-amoxiclav (875 mg/125 mg) compared to other dosing regimens and antibiotics such as clindamycin, cefaclor, cefuroxime, and ciprofloxacin. Safety and tolerability assessments indicate that co-amoxiclav is generally well-tolerated, with common mild-to-moderate gastrointestinal side effects. In summary, co-amoxiclav 1 gm remains a crucial antibiotic with optimized dosing regimens enhancing clinical outcomes while addressing resistance challenges.

## Introduction and background

Amoxicillin/clavulanate (co-amoxiclav) combines the semisynthetic penicillin amoxicillin and the β-lactamase inhibitor clavulanate potassium [1]. Oral co-amoxiclav is a key antibiotic in community healthcare settings, commonly prescribed to treat various infections. Various formulations of this medication are available for adult and pediatric patients across different regions. Co-amoxiclav is the only oral formulation combining penicillin with a β-lactamase inhibitor [2]. Notably, the clavulanic acid dose is typically limited to 125 mg in adult formulations due to tolerability issues, mainly vomiting, nausea, loose stools, and discomfort. Antibiotic-associated diarrhea due to amoxicillin-clavulanic acid treatment is the most common adverse effect, and it occurs more frequently with amoxicillin-clavulanic acid than with amoxicillin alone [3]. Despite its extensive use over the past four decades, limited pharmacokinetic/pharmacodynamic (PK/PD) studies support current dosing guidelines across various infections. Notably, clavulanate's efficacy in treating respiratory infections is scarce. The increasing prevalence of extended-spectrum beta-lactamase-producing bacteria globally raises concerns about the indiscriminate and excessive use of oral co-amoxiclav, specifically causing resistance in Gram-negative pathogens. Furthermore, disparities in susceptibility testing methods and interpretive criteria between organizations such as the Clinical and Laboratory Standards Institute and the European Committee on Antimicrobial Susceptibility Testing add complexity to treatment decisions and surveillance efforts [4].

Before 1960, the β-lactam antibiotic family consisted solely of penicillin G and penicillin V, effective against Gram-positive bacteria with a narrow spectrum of activity. In 1961, Beecham Research Laboratories (BRL) developed ampicillin, followed by amoxicillin in 1970, using the precursor 6-aminopenicillanic acid. Amoxicillin demonstrated significantly better oral absorption than ampicillin, resulting in approximately double the plasma exposure [5]. Subsequently, amoxicillin was also combined with clavulanate (the first β-lactamase inhibitor) by BRL in 1981 to tackle the emerging challenge from β-lactamase harboring *Staphylococcus aureus*, *Haemophilus influenzae, Moraxella catarrhalis*, *Escherichia coli*, *Klebsiella* spp, and *Bacteroides fragilis* [6]. The spectrum is increased to include all beta-lactamase-producing strains of the previously mentioned organisms, broadening the coverage [7].

Over the years, dosing regimens have been optimized to ensure maximal efficacy while minimizing adverse effects. The first ratio for an amoxicillin clavulanic acid combination was 4:1, as only a small amount of clavulanic acid was required due to its high affinity for inhibiting β-lactamases. Subsequently, a 7:1 ratio was introduced primarily to mitigate clavulanic acid-related toxicity [8]. Currently, formulations with ratios as high as 14:1 and 16:1 are available in certain regions. The availability of different combination ratios has allowed for tailored approaches based on geographic variations and infection profiles. In general, two principles can guide the dosing strategies of co-amoxiclav. First, to ensure adequate exposure to both co-amoxiclav, a narrower ratio of amoxicillin-clavulanic acid (typically 4:1) is preferred, administered three times daily. However, if the aim is to reduce the frequency of medication administration, a broader ratio (e.g., 7:1) can be considered, given a bid (twice a day) dosage, to enhance amoxicillin exposure while limiting clavulanic acid exposure. Such regimens are often recommended for conditions such as acute otitis media or community-acquired pneumonia (CAP) in children. Second, Gram-negative bacteria typically necessitate higher and sustained levels of both co-amoxiclav for optimal treatment efficacy. Thus, in clinical scenarios where Gram-negative pathogens are implicated, such as urinary tract infections (UTIs), a narrower ratio (e.g., 4:1) administered more frequently (three or four times daily) is warranted. Conversely, Gram-positive pathogens, with a greater affinity for clavulanic acid and susceptibility to lower amoxicillin concentrations, may suffice with wider ratio combinations (e.g., 7:1) regarding clavulanic acid exposure [8,9].

Initially, the approved dosage for adults was 250/125 mg of co-amoxiclav, administered every 8 hours. Introducing a bid dosage for convenience and compliance and addressing more severe infections became necessary. Twice daily formulations of co-amoxiclav were developed, in which the amount of amoxicillin was increased relative to clavulanate to provide equivalent bacteriological and clinical efficacy with no change in the safety profile [10]. The amoxicillin dosage was escalated to 500, 750, 875, or 1,000 mg, while the clavulanate dosage remained at 125 mg. However, increasing the clavulanate dose to 250 mg led to elevated cases of nausea without any additional enhancement in clinical efficacy [8,11,12]. The initial ratio of amoxicillin to clavulanic acid, which began as 2:1, has evolved to higher ratios such as 4:1 and 7:1. This adjustment aims to enhance antimicrobial potency and improve PK activity, especially for severe infections and resistant strains. However, the efficacy of many oral drugs has declined due to the emergence of drug-resistant *Streptococcus pneumoniae* isolates. Pharmacokinetically enhanced formulations of co-amoxiclav, such as extended-release and sustained-release, have been developed [13].

Optimizing antibiotic use is crucial for improving clinical outcomes amidst rising antimicrobial resistance and limited new drug developments [14]. Despite four decades since its introduction, co-amoxiclav remains widely prescribed. Recognizing the critical role of compliance in outpatient treatment outcomes, it is imperative to identify effective and convenient dosing regimens [1]. This review article draws significant insights from existing studies and highlights the importance of co-amoxiclav, mainly focusing on the 7:1 ratio (875 mg/125 mg) amidst rising antibiotic resistance rates and advances in PK/PD understanding.

## Review

Pharmacological properties

Mechanism of Action

Amoxicillin, a bactericidal agent in the beta-lactams class, binds to penicillin-binding proteins, inhibiting transpeptidation, and is a pivotal step in cell wall synthesis involving cross-linking. This process triggers the activation of autolytic enzymes within the bacterial cell wall, leading to cell wall lysis and subsequent bacterial cell destruction [15].

**Figure 1:**
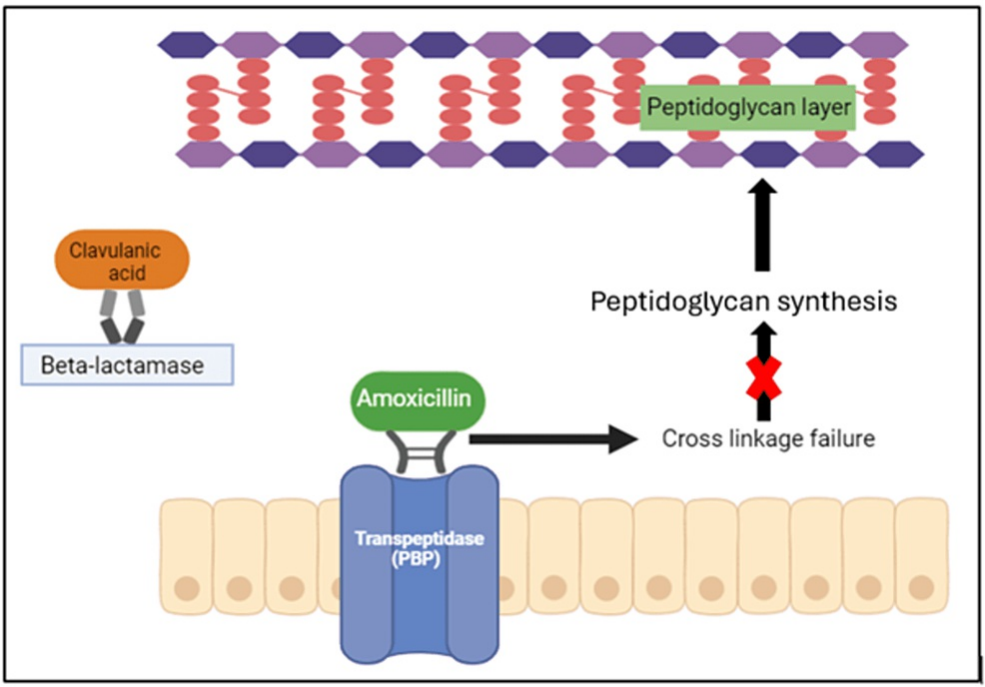
Mechanism of action of co-amoxiclav Image credit: Dr. Pal PBP, penicillin-binding protein

Clavulanic acid, often combined with amoxicillin, broadens its spectrum and has little-to-no antimicrobial activity. Over the years, certain bacteria have developed resistance to conventional beta-lactam antimicrobials due to the production of beta-lactamases. Clavulanic acid specifically targets and hydrolyzes the beta-lactam ring, which is crucial for the antimicrobial activity of penicillin-like antibiotics. It inhibits this degradation by binding to and inactivating the beta-lactamases, restoring amoxicillin's antimicrobial efficacy [7] (Figure 1).

Pharmacokinetics

Clavulanic acid and amoxicillin exhibit similar distribution patterns and have a half-life of approximately 1 hour. However, clavulanic acid differs in other PK properties. The oral bioavailability of amoxicillin is typically 70-90%, with peak serum concentrations reached 60-90 minutes after administration. In contrast, clavulanic acid demonstrates more variable oral bioavailability, averaging around 60.0 ± 23.1%. While amoxicillin is primarily excreted unchanged in the urine within 6 hours (50-80%), clavulanic acid undergoes extensive liver metabolism, with only 20-60% excreted unchanged in the urine within the same timeframe, through hydrolysis followed by decarboxylation. Both drugs have a mean elimination half-life of 1 hour each. Amoxicillin distributes well into various tissues and fluids, including the liver, lungs, prostate, muscle, middle ear effusions, maxillary sinus secretions, bone, gallbladder, bile, ascitic fluid, and synovial fluid, but penetration into cerebrospinal fluid is poor. Both drugs are widely distributed into body tissues and extracellular fluids, with low binding to plasma proteins (18-30%). Mean amoxicillin and clavulanate potassium PK parameters are shown in Table 1 [4,8,16].

**Table 1:** Mean co-amoxiclav potassium pharmacokinetic parameters Mean values were obtained from 14 normal volunteers (n = 15 for clavulanate potassium in the low-dose regimens). Peak concentrations were observed approximately 1.5 hours after dosing. The dose was administered at the start of a light meal. AUCo-24, area under the curve from time 0 to 24 hours; Cmax, peak concentration; SD, standard deviation; q8h, every 8 hours; q12h, every 12 hours

Dose and regimen (co-amoxiclav)	AUCo-24 (mcg h/mL)		Cmax (mcg/mL)	
	Amoxicillin (+ SD)	Clavulanate potassium (+SD)	Amoxicillin (+SD)	Clavulanate potassium (+SD)
250/125 mg q8h	26.7 + 4.56	12.6 + 3.25	3.3 + 1.12	1.5 + 0.70
500/125 mg q12h	33.4 + 6.76	8.6 + 1.95	6.5 + 1.41	1.8 + 0.61
500/125 mg q8h	53.4 + 8.87	15.7 + 3.86	7.2 + 2.26	2.4 + 0.83
875/125 mg q12h	53.5 + 12.31	10.2 + 3.04	11.6 + 2.78	2.2 + 0.99

Clinical efficacy studies

Co-amoxiclav (875 mg/125 mg) in Different Clinical Conditions

In two studies by Bonifaz et al., patients with cutaneous actinomycosis and actinomycetoma were treated with co-amoxiclav 875/125 mg bid. The clinical efficacy was evaluated, resulting in a clinical cure rate of 86.4% for cutaneous actinomycosis and 71% for actinomycetoma [17,18] (Table 2).

**Table 2:** Clinical efficacy studies of co-amoxiclav (875 mg/125 mg) in different clinical conditions

Trial	Study design	Sample size, study population	Intervention, dose, frequency	Results	Inference
Bonifaz et al. [18]	Long-term follow-up study	N=22 cases of cutaneous actinomycosis (91% cervicofacial, 9% abdominal)	Co-amoxiclav 875/125 mg bid for 12 weeks.	Clinical and bacteriological cure: 19/22 (86.4%) cases	Showed efficacy in treating actinomycosis with cutaneous involvement.
Bonifaz et al. [17]	Retrospective 11-year study	N=21 cases of actinomycetoma	Co-amoxiclav 875/125 mg bid	Clinical and microbiological cure: 15/21 (71%) cases	Represents an alternative or rescue treatment that has previously failed standard therapies.

Comparative Studies of Co-amoxiclav (875 mg/125 mg) Versus Other Dosing Regimens

Several comparative studies evaluated the efficacy of co-amoxiclav 875/125 mg (bid and thrice a day [tid]) versus co-amoxiclav 500/125 mg (tid) and 2000/125 mg (bid) for conditions such as lower respiratory tract infections (LRTIs), CAP, acute exacerbation of chronic bronchitis (AECB), and acute bacterial maxillary sinusitis [1,19-23]. The results indicated that the dosage regimens had comparable efficacy (Table 3).

**Table 3:** Comparative studies of co-amoxiclav (875 mg/125 mg) versus other dosing regimens AECB, acute exacerbation of chronic bronchitis; CAP, community-acquired pneumonia; LRTI, lower respiratory tract infection

Trial	Study design	Clinical condition	Sample size,	Amoxicillin/clavulanic acid dose (mg)	Clinical success rates (%)	Inference
Balgos et al. [19]	Double-blind, double-dummy study	LRTI (CAP and AECB)	324	500/125 tid	94.2	Similar efficacy
				875/125 bid	92.4	
Calver et al. [1]	Double-blind study	LRTI	557	500/125 tid	94	Similar efficacy
				875/125 bid	93	
Seggev et al. [20]	Multicenter, double-blind, randomized, double-dummy controlled trial	Acute bacterial maxillary sinusitis	170	500/125 tid	88	Similar efficacy
				875/125 bid	93	
File et al. [21]	Double-blind, randomized study	CAP	633	2,000/125 bid	90.3	Similar efficacy
				875/125 bid	87.6	
Siquier et al. [22]	Randomized, double-blind study	CAP	566	2,000/125 bid	92.4	Similar efficacy
				875/125 tid	91.2	
Sethi et al. [23]	Randomized, controlled trial	AECB	893	2,000/125 bid	93.0	Similar efficacy
				875/125 bid	91.2	

Comparative Studies of Co-amoxiclav (875 mg/125 mg) Versus Other Drugs in Different Clinical Conditions

The comparative efficacy of co-amoxiclav (875 mg/125 mg) against various drugs in diverse clinical conditions was investigated through several trials (Table 4). Comparable efficacy was observed between co-amoxiclav and clindamycin in the phase IV trial treating acute odontogenic infections [24]. Iglesias-Martín et al. found similar efficacy between co-amoxiclav and amoxicillin (1g) in preventing infections after retained third molar extraction, with co-amoxiclav showing better pain and inflammation control [25]. A randomized study revealed similar clinical response rates between cefaclor AF (advanced formulation) and co-amoxiclav in AECB [26]. Legnani et al., Cazzola et al., and Beghi et al. highlighted the superiority of co-amoxiclav over ciprofloxacin, cefixime, and azithromycin, respectively, in treating exacerbations of chronic bronchitis [27-29]. An open-label, randomized controlled trial compared the 7-day LAcR regimen (levofloxacin, co-amoxiclav, and rabeprazole) with the CAR regimen (clarithromycin, amoxicillin, and rabeprazole) in Helicobacter pylori-positive patients. LAcR showed a significantly higher eradication rate, indicating it is more effective than the standard CAR therapy [30]. Fogarty et al. showed comparable efficacy between cefditoren and co-amoxiclav in resolving clinical signs of CAP [31]. A higher clinical response rate and lower relapse rate with co-amoxiclav compared to cefuroxime axetil were found in treating chronic sinusitis [32]. In a retrospective cohort study, UTI patients treated with either co-amoxiclav or standard care showed similar clinical failure rates, but co-amoxiclav led to significantly shorter hospital stays [33].

**Table 4:** Comparative efficacy of co-amoxiclav (875 mg/125 mg) versus other drugs in various clinical conditions AECB, acute exacerbation of chronic bronchitis; AF, advanced formulation; CAP, community-acquired pneumonia; LacR, levofloxacin, co-amoxiclav, and rabeprazole; NS, not significant; UTI, urinary tract infection

Trial	Study design	Sample size, study population	Intervention, dose, frequency	Results	Inference
Dental conditions					
Tancawan et al. [24]	Phase IV, randomized, observer-blind study	N=472, odontogenic infections	Co-amoxiclav (875 mg/125 mg bid) or clindamycin (150 mg qid) for 5 or 7 days	Clinical success (%): co-amoxiclav 88.2% and clindamycin 89.7%	Similar efficacy
Iglesias-Martín et al. [25]	Comparative trial	N=546, removal of a retained third molar	Group 1, amoxicillin and clavulanate (875/125 mg) (n=257); group 2, amoxicillin (1g) (n=289)	Clinical outcomes of co-amoxiclav vs amoxicillin: pain (%) 0.55 vs 2.20 (<0.05); inflammation (%) 1.46 vs 4.76 (<0.05); trismus (%) 0.55 vs 3.84 (<0.05)	Similar efficacy
Respiratory conditions					
Bandak et al. [26]	Randomized, parallel-group, single-blind, multicenter study	N=145, AECB	7-day treatment regimens of cefaclor AF (750 mg bid, n=73) and co-amoxiclav (875/125 mg bid, n = 72)	Clinical response rate: cefaclor AF 95.9%, co-amoxiclav 97.2%	Similar efficacy
Legnani et al. [27]	Retrospective study	N=95, AECB	Co-amoxiclav (875/125 mg) 8 hours for 10 days and ciprofloxacin 500 mg 12 hours before meals for 10 days	Clinical success rate: co-amoxiclav 90%, ciprofloxacin 75.5%. Eradication rate: co-amoxiclav 84%, ciprofloxacin 57.7%	Amoxycillin-clavulanic acid showed superior clinical and microbiological efficacy
Cazzola et al. [28]	Open randomized study	N=218, AECB	N=79 with co-amoxiclav (875 mg/ 125 mg) bid, N=69 with cefixime (400 mg) once daily, and N=70 with ciprofloxacin (500 mg) bid for10 days	Eradication rate: co-amoxiclav 82.2%, cefixime 77.6%, ciprofloxacin 81.2%. Clinical success rate: co-amoxiclav 90.8%, cefixime 80.9%, ciprofloxacin 85.7%	Amoxycillin-clavulanic acid showed superior clinical efficacy
Beghi et al. [29]	Open randomized trial	N=142, AECB	Azithromycin (500 mg) OD for 3 days and co-amoxiclav (875 mg/ 125 mg) BID for 8 days	Clinical efficacy rate: azithromycin 67.6%, co-amoxiclav 97.3%. Microbiological efficacy rate: azithromycin 67.1%, co-amoxiclav 98.6%	co-amoxiclav showed superior clinical efficacy
Fogarty et al. [31]	Multicenter, prospective, randomized, investigator-blinded, parallel-group trial	N=802, CAP	Oral cefditoren 200 and 400 mg bid with oral co-amoxiclav 875/125 mg bid for 14 days	Clinical cure rates: Cefditoren 200 mg 88% cefditoren 400 mg 89.9%, co-amoxiclav 90.3%. Eradication rates: cefditoren 200 mg 84.0%, cefditoren 400 mg 88.6%, co-amoxiclav 82.6%	All three regimens showed similar efficacy
Namyslowski et al. [32]	Multicenter, open, parallel-group, randomized clinical trial	N=206, chronic or acute exacerbation of chronic sinusitis	Co-amoxiclav (875/125 mg bid for 14 days) and cefuroxime axetil (500 mg bid for 14 days)	Clinical response rate: co-amoxiclav 95%, cefuroxime 88% (p=0.07). Clinical relapse rate: co-amoxiclav 0%, cefuroxime 7% (p=0.0049)	Similar efficacy, but co-amoxiclav showed a significantly lower clinical relapse rate
Gastrointestinal conditions					
Chen et al. [30]	Open-labeled, prospective, randomized, and controlled study	N=153, Helicobacter pylori-positive, therapy-naïve patients with a positive rapid urease test	Levofloxacin (500 mg), co-amoxiclav (875 mg/125 mg), and rabeprazole (20 mg) bid (LAcR) or clarithromycin (500 mg), amoxicillin (1000 mg), and rabeprazole (20 mg) bid (CAR) for 7 days	Helicobacter pylori eradication rates: LAcR 78.1%, CAR- 57.5% (p=0.008)	LAcR regimen showed higher Helicobacter pylori eradication efficacy when compared with the standard therapy
Urinary tract infection					
Salam et al. [33]	Retrospective cohort study	N=50	Co-amoxiclav 875 /125 mg every 12 hours for 7 days vs standard of care group (ertapenem, meropenem, trimethoprim/sulfamethoxazole, levofloxacin, amikacin, piperacillin/tazobactam, or nitrofuran)	Clinical failure rate within 90 days after therapy: co-amoxiclav 19.2%, standard of care 30.3% (p=0.332). Hospital length of stay in days (admitted for community): co-amoxiclav 2.9, standard of care- 8.4 (p=0.053)	Similar clinical failure rates, but co-amoxiclav had a shorter duration of stay

Safety and tolerability

Co-amoxiclav is generally well tolerated, with a low incidence of adverse events. Several studies showed no statistically significant difference in the adverse event rates [28,31] (Table 5). Common mild-to-moderate gastrointestinal side effects include nausea, vomiting, cramping (prevalence 3-6%), or diarrhea (4-15%). To mitigate clavulanic acid-related toxicity, a 7:1 ratio was introduced [8]. Additionally, Bax reviewed clinical trials that indicated a slight decrease in the frequency of diarrhea when comparing the bid regimen to the tid regimen [10]. In a long-term follow-up study, patients with actinomycosis treated with co-amoxiclav reported mild nausea and diarrhea, which did not necessitate further treatment [18]. A phase IV randomized trial between co-amoxiclav and clindamycin showed a comparable safety report consistent with the known pharmacologic effects of co-amoxiclav [24].

Allergic reactions to β-lactams occur in 0.7-8% of patients receiving treatment [34]. While transient increases in serum transaminases are typical following co-amoxiclav therapy, hepatic injuries are uncommon, with an incidence rate ranging from 1 to 1.7 per 10,000 users. Co-amoxiclav-induced hepatitis is generally reversible upon discontinuation of the drug, although the duration of symptoms can vary widely, lasting from one to eight weeks [16].

**Table 5:** Safety analysis of co-amoxiclav (875 mg/125 mg) versus other drugs in various clinical studies AE, adverse event; AECB, acute exacerbation of chronic bronchitis; BUN, blood urea nitrogen; CAP, community-acquired pneumonia; TEAEs, treatment-emergent adverse effects

Trial	Study design	Sample size, study population	Intervention, dose, frequency	Results	Inference
Bonifaz et al. [18]	Long-term follow-up study	N=22, cutaneous actinomycosis	Co-amoxiclav 875/125 mg bid for 12 weeks.	AE: 4/22 cases (18.2%), of which three reported nausea and one reported diarrhea	The events were mild and did not require additional treatment.
Tancawan et al. [24]	Phase IV, randomized, observer-blind study	N=472, odontogenic infections	Co-amoxiclav (875 mg/125 mg bid) or clindamycin (150 mg qid) for 5 or 7 days	Number of TEAEs: co-amoxiclav 243, clindamycin 236. Diarrhea: co-amoxiclav 8.1%, clindamycin-11.9%	The overall incidences of TEAEs were similar across both the treatment arms.
Cazzola et al. [28]	Open randomized study	N=218, AECB	N=79, co-amoxiclav 875/125 mg bid N=69 with cefixime (400 mg) OD, N=70 with ciprofloxacin (500 mg) bid for an average period of 10 days	Adverse event rate: co-amoxiclav 8.9%, cefixime 14.7%, ciprofloxacin 12.9%	No statistically significant differences among the three treatment groups.
Beghi et al. [29]	Open randomized trial	N=142, AECB	Azithromycin (500 mg) OD for 3 days and co-amoxiclav 875/125 mg bid for 8 days	Number of AEs: co-amoxiclav 1 (diarrhea), azithromycin 2, (epigastralgia and alterations in BUN, creatinine)	Both groups were well tolerated
Fogarty et al. [31]	Randomized, investigator-blinded, parallel-group trial	N=802, CAP	Oral cefditoren 200 and 400 mg bid with oral co-amoxiclav 875/125 mg bid for 14 days	AE incidence: Cefditoren 200 mg 38%, cefditoren 400 mg 35%, co-amoxiclav 39%. Number of diarrheal events: cefditoren 200 mg 14%, cefditoren 400 mg 21%, co-amoxiclav 21%	Both groups were well tolerated.

Dosage and administration

Co-amoxiclav in the 875 mg/125 mg dosage has been effectively employed in the treatment of a wide array of infections, including LRTI, ENT infections, skin and soft tissue infections, dental infections, UTI and gastrointestinal infections, and severe infections [1,4,18-21,23-25,30,33]. Co-amoxiclav demonstrates excellent absorption in the gastrointestinal tract after administering tablet formulations, regardless of meals [8]. Patients with impaired renal function do not generally require a dose reduction unless the impairment is severe. Specifically, patients with a glomerular filtration rate (GFR) of less than 30 mL/min should not receive the 875 mg dose [35].

## Conclusions

In conclusion, this narrative review emphasizes the pivotal role of co-amoxiclav 875 mg/125 mg, a 7:1 ratio, in managing various bacterial infections. The comprehensive exploration of its pharmacology, mechanisms of action, spectrum of activity, and clinical evidence affirms its central position as a fundamental antibiotic in modern medical practice. Clavulanic acid and amoxicillin have a similar half-life of about one hour and a Cmax of 11.6 ± 2.78 mcg/mL. They are widely distributed in body tissues and fluids, including the liver, lungs, prostate, muscles, and synovial fluid, with low plasma protein binding. The pharmacodynamics of co-amoxiclav highlight its efficacy in inhibiting bacterial cell wall synthesis and overcoming beta-lactamase-mediated resistance. The dosing regimen has been refined to maximize efficacy and minimize adverse effects, and the twice-daily dosage has been adopted for convenience and compliance. This ratio aims to amplify antimicrobial potency, addressing more severe infections and resistant strains. As clinicians navigate the complexities of infectious diseases, the insights from this review offer guidance on evidence-based decision-making and optimizing patient outcomes.
